# Assessing the impact adenotonsilectomy has on the lives of children with hypertrophy of palatine and pharyngeal tonsils

**DOI:** 10.1016/S1808-8694(15)30833-8

**Published:** 2015-10-18

**Authors:** Bernard Soccol Beraldin, Tatiana Rocha Rayes, Paulo Henrique Villela, Denise Marchi Ranieri

**Affiliations:** 1 Student.; 2 Medical Student - UNIVALI, SC.; 3 MSc. Professor of Otolaryngology - UNIVALI, SC.; 4 MSc. Professor of Otolaryngology - UNIVALI, SC.; Universidade Do Vale Do Itajaí, Santa Catarina.

**Keywords:** adenoids, children, quality of life

## Abstract

The hypertrophy of the palatine and pharyngeal tonsils is extremely common in children, being one of the most frequent causes of visits to otolaryngologists and such problem can impair the child’s quality of life. **Aim:** to evaluate the impact of adenotonsillectomy on the lives of children with hypertrophied tonsils. **Materials and methods:** Contemporary longitudinal cohort study. A specific questionnaire used to evaluate the quality of life - OSD-6, was given to seventy five parents or guardians of children previously submitted to adenotonsillectomy, before the surgery and thirty days afterwards. **Results:** The adenotonsillectomy provided a significant reduction in the questionnaire score. **Discussion:** Snoring and nasal obstruction were the symptoms with the highest scores. There is a great concern from the parents with the snoring of the children and a poor statistic correlation between the degree of obstruction degree and a worse quality of life. **Conclusion:** Adenotonsillectomy causes a relevant impact in the quality of life of children with tonsil hypertrophy.

## INTRODUCTION

One of the most frequent disorders encountered in the otolaryngologist’s office is the enlargement of palatine and pharyngeal tonsils. It is the main cause of sleep disorders in children, and responsible for 75% of cases of sleep apnea^1^. Surgical removal of the tonsils is the treatment of choice, being of the procedure more commonly performed by otolaryngologists^2^.

Respiratory disorders during sleep are entities known in the medical field and has been constantly studied because of its high prevalence and important morbimor-tality. Since it is such a common occurrence, often times it is not considered a disease and, therefore, is not reported in routine puericulture papers^3^, ^4^. Not treating children with this disorder can bring about a number of problems: low school performance, non-specific behavior disorders, lack of attention, development delays, problems with mastication and swallowing, oral breathing and its craniofacial repercussions, day sleepiness, enuresis, cor pulmonale and height-weight deficit^5^, ^6^.

Symptoms and complications caused by upper airway obstruction lead to a significant reduction in the quality of life of these patients^7^. There are some treatment possibilities such as CPAP (Continuous Positive Airway Pressure) and weight loss in obese patients; however, these alternatives are not well tolerated by children and are rarely adopted as primary treatment^8^. Despite the controversies between otolaryngologists and pediatricians, adenotonsillectomy is curative for the majority of patients^9^. Indications for this surgical procedure are well established; however, there are only a few studies about its impact on the patient’s quality of life.

The physical consequences in general, represent the greatest concerns physicians have regarding the disease’s impact^7^. Nonetheless, the disease impact on the quality of life of patients and their guardians must also be taken into account when treatment choice is being considered.

When we talk about public health, there is the need to guide treatment decision as to the best deployment of resources from the health care system. Thus, it is important to stress life quality impairment of a given pathology so as to show its importance for the individual at a social level or at a health care level within a community.

Having discussed the aforementioned remarks, the present study aims at assessing the impact adenotonsillectomy has on the quality of life of children with enlarged pharyngeal and palatine tonsils, by comparing the results obtained from a specific questionnaire about quality of life, employed before and after the surgical procedure.

## MATERIALS AND METHODS

Contemporary longitudinal cohort study carried out through a life quality questionnaire with children with pharyngeal and palatine tonsil enlargement, OSD-6, before and after adenotonsillectomy was performed.

The population was submitted to the study from October 2005 to August 2006. 75 children with adenotonsillectomy indication because of pharyngeal tonsil hyperplasia obstructing over 50% of the rhinopharynx (based on cavum radiography findings), and palatine tonsils enlargement (grade II or more seen through the physical exam), associated with sleep disorders and respiratory complaints. Patients from both genders were included, from 3 to 14 years of age.

**Figure 1:**
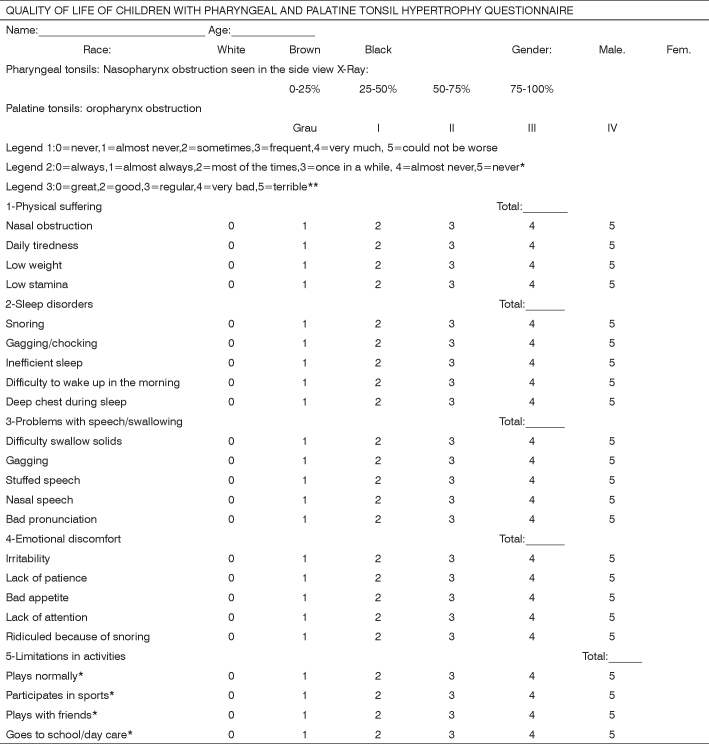
Model of questionnaire about quality of life^7^.

The patients’ parents or guardians were interviewed by means of a specific questionnaire - OSD-6, based on the work of Di Fransesco et al.^7^ (Figure 1), one day before and one month after the surgery, after they were duly informed about the study and signed the Free and Informed Consent. Such questionnaire included the following domains: physical suffering (1), sleep disorder (2), speech and swallowing disorders (3), emotional discomfort (4), physical activity limitations (5) and concerns from parents and guardians about the child’s snoring (6) and there are three legends that serve to quantify each item on the domains, in other words, how the guardians believe the symptoms affect their children. The first (0=never, 1=almost never, 2=sometimes, 3=frequently, 4=very much, 5=could not have been worse) can be used for all domains, except the fifth. The second (0=always, 1=almost always, 2=most of the times, 3=once in a while, 4=almost never, 5=never) for the first four items of the fifth domain, and the third (0=great, 1=good, 2=regular, 3=bad, 4=very bad, 5=terrible) for the last item of the same domain. Thus, the higher the score obtained in the questionnaire, the worse is the child’s quality of life. Since the maximum score for each item is five and the number of patients interviewed was 40, the domain already has four items (1) that may yield a maximum score of 1,500 points, now, the domains which have five items (2, 3, 4 and 5) produce 1,875 points and the last domain (6) 375 points, since it does not have items.

Patients with craniofacial, neurologic disorders, those with surgery indication only due to recurrent tonsillitis and the patients who would be submitted to some other surgical intervention on the same date were taken off the study. None of the guardians refused to participate in this study.

We compared the scores obtained before and after the adenotonsillectomy, establishing whether or not there is improvement in the quality of life of these children after the surgery, checking which were the domains that got the highest score and among them, which were the items responsible for that score. In order to check whether or not there was a relationship between the degree of tonsilar obstruction and a worse quality of life and if there is a correlation among the questionnaire domains, we tested the hypothesis of a simple correlation among the questionnaire’s domains, p ≤ 0.05 by means of the Spearman’s method.

After the end of the study, the results were given to those in charge of the research and the interviewees who were interested in them.

The current study was delivered to the Ethics in Research Committee in September of 2006 and was approved in October of the same year, under protocol # 448/2005.

## RESULTS

We interviewed 75 guardians of patients, with ages varying between 3 and 14 years (mean of 7.05), 30 (40%) were males and 45 (60%) females, 18 (24%) were brown, 11 (15%) back and 46 (61%) white. As to the pharyngeal tonsil, 33 (45%) and 42 (55%) had nasal-pharyngeal obstructions that varied between 50 and 75% and 75 and 100%, seen through cavum radiography, respectively. Seven (10%) patients had level II obstruction of the oropharynx through the physical exam, 12 (15%) level III obstruction and 56 (75%) had level IV obstruction.

On Tables 1 and 2 we see the scores obtained in each domain before and after adenotonsillectomy, respectively. We observed that the parents/guardians concern with the child’s snoring was the domain that, proportionally, obtained the highest score (78%) in the preoperative period, followed by physical suffering (43%) and sleep disorders (41.5%). Now, the domain associated with activity limitation was the one with the lowest score, obtaining only 140 (7.5%) points from a total of 1,875 possible.

**Table 1:** Score obtained in the pre-adenotonsillectomy period.

Domain	N	Minimum	Summation	Maximum	%
Physical suffering	75	0	645	1500	43,0%
Sleep disorder	75	0	778	1875	41,5%
Speech and swallowing problems	75	0	645	1875	34,4%
Emotional discomfort	75	0	656	1875	35%
Activity limitation	75	0	140	1875	7,5%
Parents’ concern with the snoring	75	0	292	375	78,0%
Summation of all the domains		0	3156	9375	33,,6%

**Table 2:** Score obtained in the post-adenotonsillectomy domains.

Domain	N 75	Minimum	Summation	Maximum	%
Physical suffering	75	0	112	1500	7,5%
Sleep disorder	75	0	125	1875	6,7%
Speech and swallowing problems	75	0	120	1875	6,4%
Emotional discomfort	75	0	262	1875	14,0%
Activity limitation	75	0	116	1875	6,1%
Parents’ concern with the snoring	75	0	8	375	2,0%
Summation of all the domains		0	743	9375	7,9%

On Table 2 we see that all the domains reduced significantly the score percentage obtained, except for the activity limitation domain, which kept almost the same percentage obtained in the preoperative (6.1%).

On Table 3 we see the variations among pre and postoperative percentages. The domain of parents’ concern with the child’s snoring was the most significant one (76%), followed by physical suffering (35.5%) and sleep disorder (34.0%). The emotional discomfort had the second lowest difference (21.0%), behind only that of activity limitations (1.4%). In relation to the summation of all the domains, the difference was of 25.7%.

**Table 3:** Percentage values before and after adenotonsillectomy.

Domain	Pre-op.	Post-op.	Variation
Physical suffering	43,0%	7,5%	35,5%
Sleep disorder	41,5%	6,7%	34,0%
Speech and swallowing problems	34,4%	6,4%	28,0%
Emotional discomfort	35%	14,0%	21,0%
Activity limitation	7,5%	6,1%	1,4%
Parents’ concern with the snoring	78%	2%	76,0%
Summation of all the domains	33,6%	7,9%	25,7%

Table 4 shows the item from each domain that obtained the highest preoperative score and the score obtained after surgery. The items that added the most points after adenotonsillectomy were: nasal obstruction (275), snoring (307), nasal voice (172), lack of patience (180) and school and day-care attendance (58). All had low postoperative score, except the item associated with school and day-care attendance which kept the same score.

**Table 4:** Items from the domain that got the highest scores.

Items	Pre-op.	Post-op.
Nasal obstruction	275	22
Snoring	307	18
Nasal voice	172	31
Lack of patience	180	97
School and day-care attendance	58	58
Parents’ concern with snoring	290	5

**Figure 2:**
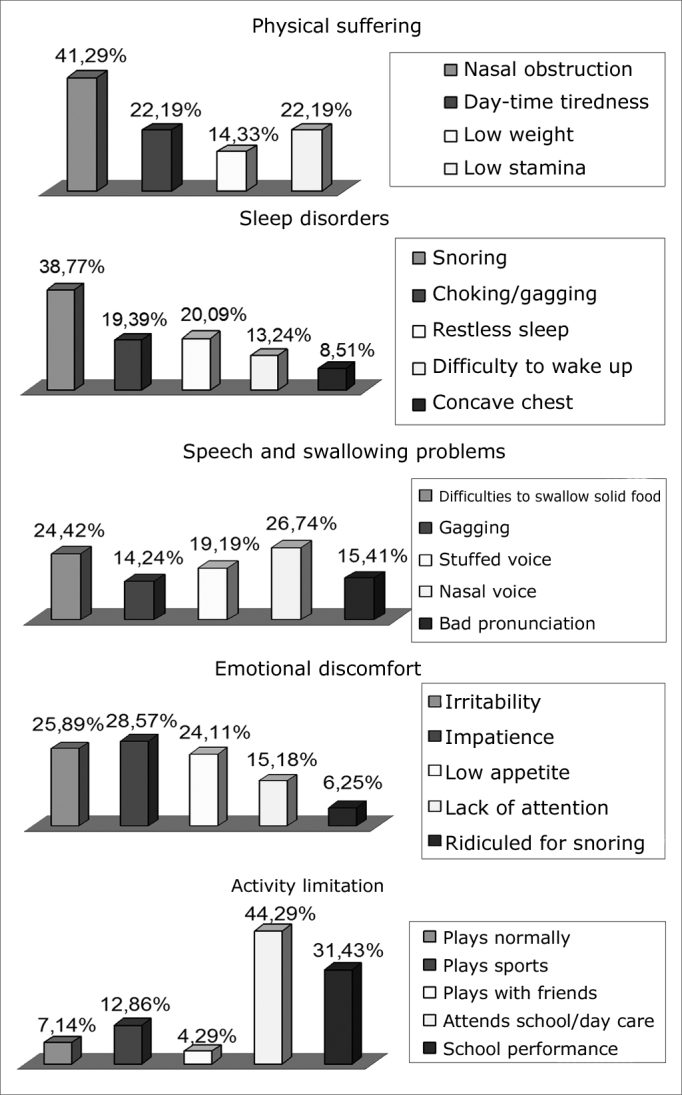
Distribution of the percentages from items of the pre-adenotonsillectomy domains.

These items were responsible for 41.29%, 38.77%, 26.74%, 28.57% and 44.29% of all the domains before the surgery (Fig. 2).

As we correlate the level of palatine tonsil obstruction with the domains, we found a statistically positive and significant correlation between the level of obstruction and physical suffering, speech and swallowing problems and the summation of all the domains. Now, as we correlate the level of pharyngeal tonsil obstruction in the same fashion, we found a correlation with physical suffering, sleep disorders and parents’ concern with the child’s snoring (Table 5).

**Table 5:** Correlation between the level of tonsil obstruction and the domains.

	Palatine tonsils		Pharyngeal tonsils	
Physical suffering	p coefficient		p coefficient	
Sleep disorders	0.45230	0.0034	0.43922	0.0046
Speech and swallowing problems	0.18398	0.2558	0.29135	0.0482
Emotional discomfort	0.31241	0.0497	0.05239	0.7482
Activities limitations	0.02754	0.8661	0.10497	0.5192
Parents’ concern with snoring	0.15275	0.3467	0.27733	0.0832
Summation of all the domains	0.06596	0.6860	0.31153	0.0204
Soma de todos os dominios	0.37358	0.0176	0.28328	0.0765

Spearman’s non-parametric correlation (n = 40 p ≤ 0.05).

## DISCUSSION

Historically, adenotonsillectomy has been the most commonly performed surgical procedure in otorhinolaryngology, mainly involving the pediatric population^10^. Thus, most children will suffer their first surgical intervention within otorhinolaryngology.

Physicians tend to be concerned only with the physical consequences of diseases, and with the laboratorial repercussions that such affection can cause.

Recently, quality of life has been used to try to assess the impact diseases have on their patients and guardians, adding such consideration to the treatment decision process^10^, ^11^, ^12^, ^13^, ^14^, ^15^. The questionnaire used in this study is a specific instrument and aims at assessing the impact a disease may have on a patient, in an attempt to incorporate such criterion to the medical indications for severity and importance of assessing children with tonsilar enlargement and to add such fact to the treatment decision process.

Disease-specific questionnaires such as this one focus on areas or functions which belong to particular diseases or conditions and are used to describe the impact diseases have on individuals, going beyond the usual morbidity investigation patterns used to assess the severity of a certain particular disease or condition^16^.

In our study, 100% of the children submitted to the surgical procedure had a score reduction in all the domains, in other words, life quality improvement showing the benefits of adenotonsillectomy in children with tonsil hypertrophy, respiratory complaints and sleep disorders.

Parents’ concern with the child’s snoring; physical suffering and sleep disorders were the domains that achieved the highest scores in the pre-adenotonsillectomy questionnaire. These are the same domains that stood out in a similar paper published by Serres et al.^11^; however, the author also firstly found sleep disorders, followed by physical suffering and parents’ concern with the child’s snoring, which may have occurred because of cultural differences. Analyzing the score obtained after surgery in these domains, we notice that they keep the same order, in other words, parents’ concern was the domain which obtained the highest percentage difference, afterwards was physical suffering and finally, sleep disorders. Thus, the domains responsible for the worst quality of life (highest score) for these children were the ones that most improved after adenotonsillectomy.

The activity limitation domain was the one that scored the lowest in the preoperative period, coinciding with the findings from Serres et al.^11^. Its percentage variation in relation to the post-adenotonsillectomy score was also the one with the lowest score, only 1.4%. This can be explained by the fact that the parents’ concern was more often associated with physical problems and sleep disorders because they were deemed of having the highest risk for health or because of the fact that a volume increase in tonsils is not able to limit the daily activities of children, at least among the ones included in our study.

When we analyze the items of each domain separately in order to know which of them was responsible for the highest score, we found: nasal obstruction, snoring, nasal voice, lack of patience and school or day-care attendance with the highest percentages associated with their specific domains. We did not find similar data in the literature for possible comparisons.

In relation to the nasal obstruction items and snoring, they were responsible for 41.29% and 38.77% of the total of their domains. This represents almost twice the item that had the second highest score in its respective domains, which were daily tiredness and bad stamina, both with 22.19% in the physical domain and non-effective sleep. Such fact shows the extreme attention that parents pay regarding the children’s lack of capacity to breathe through the nose (oral breathing) and with noisy breathing during sleep, and such fact can be decisive for treatment indication.

As we correlate the level of tonsilar obstruction with the score obtained in the questionnaire domains, we noticed that there is an association between physical sufferings with both tonsils. However, a larger palatine tonsil volume is still associated with speech and swallowing disorders and domain summation, while pharyngeal tonsils were associated with sleep disorders and parents’ concern. Although this correlation exists, it is weak. Such fact is similar to the findings by Di Fransesco et al.^7^ who besides correlating the obstruction level of the upper airways, also correlated craniofacial morphological alterations and concluded that both factors were not responsible for a worse quality of life.

## CONCLUSION

Adenotonsillectomy causes a positive impact, in other words, a significant improvement in the quality of life of children with tonsil hypertrophy.
